# Supplementary material for: Identification of photoperiod-induced specific miRNAs in the adrenal glands of Sunite sheep (Ovis aries)
Source: Front Vet Sci. 2022 Jul 22;9:888207. doi: 10.3389/fvets.2022.888207 (PMC9354845; doi:10.3389/fvets.2022.888207)
Supplement: Supplementary file 1 [file Data_Sheet_1.ZIP › Supplementary file/miRDeep2 file.zip!/miRDeep2 file/Novel_Structure/SP42A3/Novel_458.pdf]

Provisional ID	: NC_019460.2_7736
Score total	: 2.1
Score for star read(s)	: -1.3
Score for read counts	: 0
Score for mfe	: 1.8
Score for randfold	: 1.6
Score for cons. seed	:
Total read count	: 20
Mature read count	: 20
Loop read count	: 0
Star read count	: 0

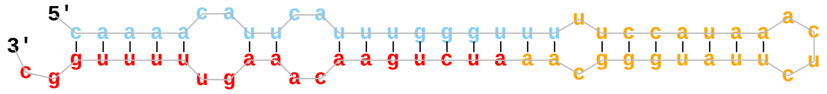

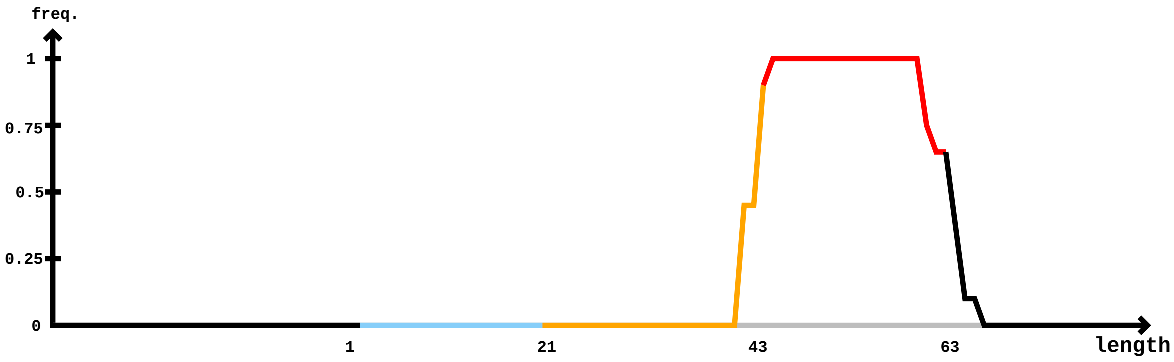

Star Mature

```
5'- auaaaacauggaugaaaauauuggguuggccaaaacauucauuuggguuuuuccauaaacucuuaugggcaaaucugaacaaaguuuuuggccaacucaguguuaugcuaaa   -3'    exp
    ................(((((((((((((((((((..((..(((((((((.(((((((....))))))).)))))))))..))..)))))))))))))))))))........    reads    mm    sample
    ......................................................................aaaucugaacaaaCuuuuu.......................    5        1     seq
    ......................................................................aaaucugaacaaaCuuuuug......................    2        1     seq
    ......................................................................aaaucugaacaaaguuuuuggc....................    1        0     seq
    ......................................................................aaaucugaacaaaCuuuuuggcc...................    1        1     seq
    ........................................................................aucugaacaaaCuuuuuggc....................    9        1     seq
    .........................................................................ucugaacaaaCuuuuuggccaa.................    2        1     seq
```
